# How Age-Friendly Is Your City to You: Experiences From a Mid-Sized German City

**DOI:** 10.1093/geroni/igaf122.1196

**Published:** 2025-12-31

**Authors:** Adele Grenz, Michael Weinhardt, Moritz Hess, Joost van Hoof, Jeroen Dikken, Kathrin Boerner

**Affiliations:** The Carl von Ossietzky University of Oldenburg’s School of Medicine and Health Sciences, Oldenburg, Niedersachsen, Germany; German Centre of Gerontology, Berlin, Berlin, Germany; University of Applied Sciences Niederrhein, Krefeld, Nordrhein-Westfalen, Germany; The Hague University of Applied Sciences, Wrocław University of Environmental and Life Sciences, The Hague, Zuid-Holland, Netherlands; The Hague University of Applied Sciences, The Hague, Zuid-Holland, Netherlands; Carl von Ossietzky University of Oldenburg, Oldenburg, Niedersachsen, Germany

## Abstract

Urbanization and demographic change pose social challenges, underscoring the need for a systematic approach to assessing the age-friendliness of urban environments and the experiences of older adults within them. The purpose of this study was to provide a valid German version of the Age-Friendly Cities and Communities Questionnaire (AFCCQ), which aligns with the WHO’s eight domains of age-friendliness, and to examine personal and contextual correlates of perceived age-friendliness in a mid-sized city. A representative sample of older adults (N = 905) assessed their city’s age-friendliness, with average scores reflecting at least moderate satisfaction in all domains, but also significant interindividual variation. A cluster analysis identified several distinct groups, including a group of rather vulnerable individuals facing health challenges and socioeconomic disadvantage — who reported the lowest age-friendliness scores — as well as a more affluent, healthier residents who rated their environment positively across all domains. Late life phase was also a factor, with very old adults overrepresented in the less satisfied groups. Narrative responses to open-ended follow-up questions specifically highlighted challenges in mobility, healthcare access, and caregiving support. Findings indicate that health conditions, living arrangements and socio-economic status may critically shape how older adults experience urban environments. These results emphasize the need for targeted policies to ensure urban environments are inclusive and accessible for all older adults. Additionally, this study underscores the value of systematic evaluations in shaping and monitoring urban policies that align with age-friendly principles.

